# Impact of pericoronary adipose tissue attenuation on recurrence after radiofrequency catheter ablation for atrial fibrillation

**DOI:** 10.1002/clc.24081

**Published:** 2023-07-12

**Authors:** Zhe Wang, Yi‐Jia Wang, Jia‐Wei Chen, Li‐Chen Ren, He‐He Guo, Xiao‐Jie Chen, Jian‐Zeng Dong, Ying‐Wei Chen, Yi‐Hong Sun

**Affiliations:** ^1^ Department of Cardiology, China‐Japan Friendship Hospital Chinese Academy of Medical Sciences & Peking Union Medical College Beijing China; ^2^ Department of Cardiology, Beijing Hospital Chinese Academy of Medical Sciences & Peking Union Medical College Beijing China; ^3^ Department of Cardiology The First Affiliated Hospital of Zhengzhou University Zhengzhou Henan China; ^4^ Department of Radiology The First Affiliated Hospital of Zhengzhou University Zhengzhou China; ^5^ Department of Cardiology Anzhen Hospital Affiliated to Capital Medical University Beijing China; ^6^ Department of Cardiology China‐Japan Friendship Hospital Beijing China

**Keywords:** atrial fibrillation, pericoronary adipose tissue attenuation, radiofrequency ablation, recurrence

## Abstract

**Background:**

Inflammation plays a vital role in the occurrence and progression of atrial fibrillation (AF). The association between pericoronary adipose tissue attenuation (PCATA) and AF recurrence following ablation has not been fully clarified.

**Hypothesis:**

We aimed to evaluate the association between PCATA and AF recurrence after radiofrequency catheter ablation (RFCA).

**Methods:**

Patients who underwent the first RFCA for AF and performed coronary computed tomography angiography before ablation between 2018 and 2021 were enrolled. The predictive values of PCATA for AF recurrence after ablation were investigated. The area under curve (AUC), relative integrated discrimination improvement (IDI), and categorical free net reclassification improvement (NRI) were used to assess the discrimination ability of different models for AF recurrence.

**Results:**

During 1‐year follow‐up, 34.1% patients experienced AF recurrence. The multivariable analysis model revealed that PCATA of the right coronary artery (RCA) was an independent risk factor for AF recurrence. Patients with a high level of RCA‐PCATA had a high risk of recurrence, after adjusting for other risk factors by restricted cubic splines. The performance in predicting AF recurrence was significantly improved by adding the marker of RCA‐PCATA to the clinical model (AUC: 0.724 vs. 0.686, *p* = .024), with a relative IDI of 0.043 (*p* = .006) and continuous NRI of 0.521 (*p* < .001).

**Conclusions:**

PCATA of RCA was independently associated with AF recurrence after ablation. PCATA may be helpful for risk classification for AF ablation patients.

## Graphical abstract

- The high level of right coronary artery‐pericoronary adipose tissue attenuation (RCA‐PCATA) is an independent predictor for atrial fibrillation (AF) recurrence after radiofrequency ablation. PCATA surrounding the proximal RCA may identify high‐risk recurrence in AF ablation patients.

- The PCATA index in coronary computed tomography angiography reporting might be more meaningful for assessing AF risk stratification following ablation.

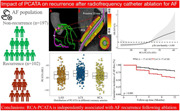

## 1 INTRODUCTION

Atrial fibrillation (AF) is the most prevalent cardiac arrhythmia and increased dramatically over the past 30 years.^1^ Radiofrequency catheter ablation (RFCA) is a major treatment modality for patients with AF.^2^, ^3^ Approximately 70%−90% of paroxysmal and 65%−75% of persistent AF without atrial arrhythmia appears within 1 year after ablation.^4^ There has been a heightened emphasis on risk factors modification in reducing the AF recurrence following ablation.^5^

Inflammation plays a vital role in the initiation and maintenance of AF. The electrophysiology and structural properties of the atria are critically affected by inflammatory processes.^6^ The fat attenuation index has been demonstrated to be associated with 18^F^‐fluorodeoxyglucose uptake on positron emission tomography and adipocyte lipid content quantified by histology, indicating that the fat attenuation index could reflect perivascular inflammation.^7^ One study found that the attenuation of periatrial adipose around the left atrial (LA) is independently associated with AF recurrence following ablation.^8^ The pericoronary adipose tissue attenuation (PCATA) is proven to be strongly associated with inflammatory changes.^7^ The association between PCATA and the recurrence of AF after RFCA has not been clear. This study aims to investigate the association between PCATA and AF recurrence after RFCA.

## 2 METHODS

### 2.1 Study design and populations

We conducted a retrospective study of AF patients who received the first catheter ablation from January 2018 to April 2021 in the First Affiliated Hospital of Zhengzhou University. The inclusion criteria were: (1) patients underwent coronary computed tomography angiography (CCTA) before ablation. (2) Patients without history of coronary heart disease. The exclusion criteria were patients with pacemaker implantation, thyroid dysfunction, renal dialysis, advanced valvular heart disease, hypertrophic cardiomyopathy, and patients with poor/insufficient CCTA images. Patients who were dead or lost to follow‐up were also excluded. The study complied with the Declaration of Helsinki. The study protocol was authorized by the local institution's ethics committee, and waived the need for written informed consent.

### 2.2 Clinical and laboratory data

The following data were collected from hospital records: demographic parameters, comorbidities, echocardiography parameters, CCTA parameters, and medications on admission. The duration of AF was calculated by the time from the date of initial symptom onset or first diagnosis of AF to the RFCA index date.

### 2.3 CCTA acquisition

All CCTA scanning was accomplished using dual‐source computed tomography (CT) of a third‐generation scanner (Somatom Force; Siemens Heathineers). CCTA images were acquired with the following scan protocol: Tube voltage was 100 or 120 kV. If 100 kV voltage was used instead of 120 kV voltage, the mean Hounsfield units (HU) value was corrected dividing by 1.11485, as previously reported.^9^, ^10^ If the patient's heart rate was >65 beats/min, an oral or intravenous beta‐blocker was administered to reduce the heart rate before scanning. Sublingual nitroglycerine (0.5 mg) was also administered immediately before CCTA scanning. Images were acquired after a bolus injection of 30−60 mL of contrast media (400 mg iodine per mL, Iomeprol Injection). Using prospective ECG‐triggering with automatic tube current modulation, field matrix of 512 × 512, and scan slice thickness of 0.5 mm. All data were transferred to the reconstructed workstation for analysis.

### 2.4 Epicardial adipose tissue (EAT) volume assessment

EAT is a low‐density margin encasing the myocardium in the pericardial space on CCTA. EAT was defined as CT density ranging from −190 to –30 HU.^11^ EAT volume was quantified using dedicated semiautomatic software (SyngoVia; Coronary Plaque Analysis; version 5.0.1; Siemens Healthineers), as shown in Supporting Information: Figure S1. The volume of EAT was automatically delineated and calculated with the software and adjusted manually if necessary.

### 2.5 The measurement of PCATA

**Figure 1:**
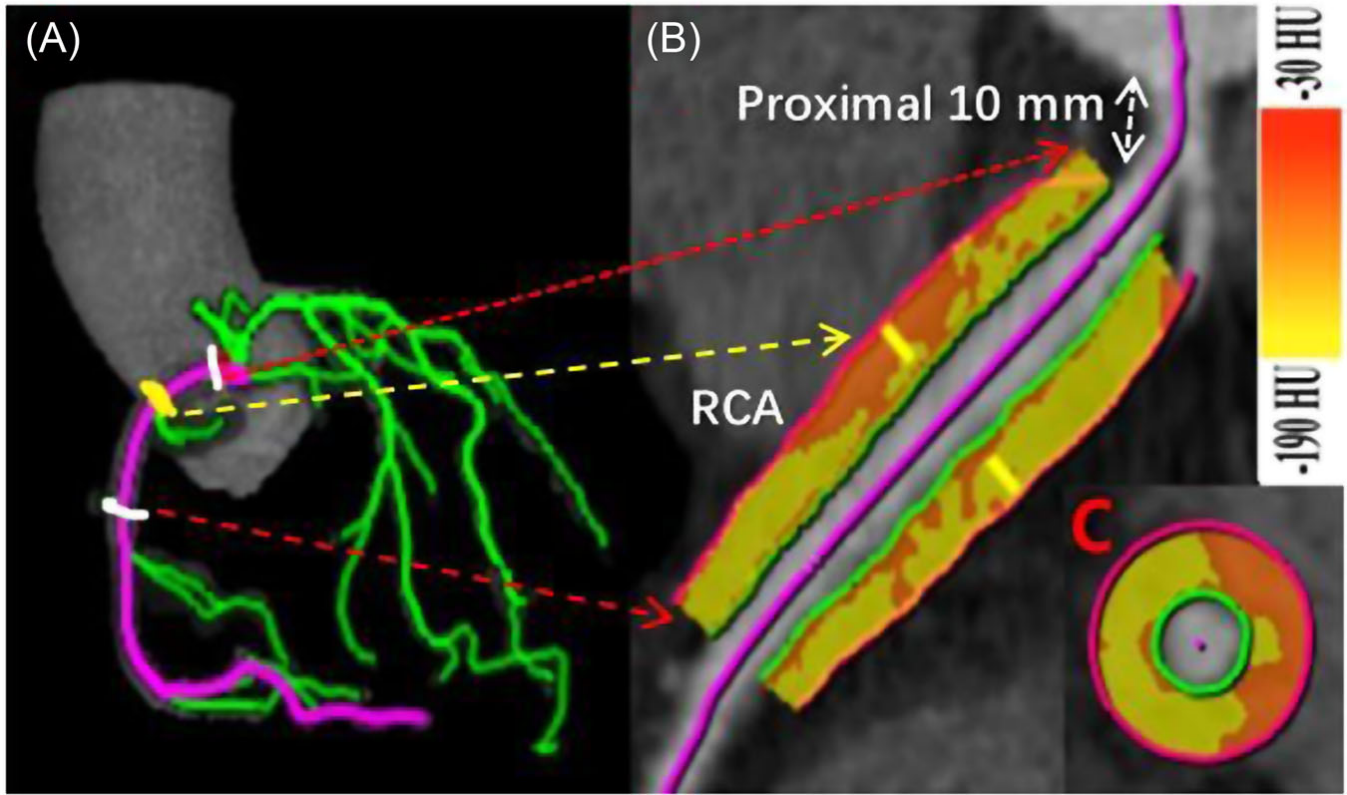
Semiautomated PCATA of RCA analysis on coronary computed tomography (CT) angiography. (A) Three‐dimensional reconstruction of all three major epicardial coronary vessels. (B) The PCAT phenotyping around the proximal RCA 10−50 mm segment was traced for the vessel. (C) The color map of coronary CT of PCATA in the cross‐sectional view. PCAT was defined as fat within a radial distance equal to the diameter of the vessel. AF, atrial fibrillation; HU, Hounsfield unit; PCAT, pericoronary adipose tissue; PCATA, pericoronary adipose tissue attenuation; RCA, right coronary artery.

For each patient, we measured the value of PCATA by the dedicated workstation (SyngoVia; Coronary Plaque Analysis; version 5.0.1; Siemens Healthineers). The weighted mean CT attenuation of PCAT (voxels with attenuation between −190 and −30 HU) within the range of the distance from the outer vascular wall, which is equal to the average diameter of the targeted vessel, was defined as PCATA.^12^ According to the previous study, the proximal segment of the three main coronary artery branches with a length of 40 mm was selected for PCATA measurement. To avoid the influence of the aortic wall, a 10 mm proximal segment of the right coronary artery (RCA) was excluded, and a 10−50 mm proximal segment of the target vessel was selected, as shown in Figure 1. The left main coronary artery was not analyzed due to its large length variability.^12^ The PCATA analysis was performed in the proximal 40 mm segments of in the left anterior descending (LAD) and left circumflex (LCX), as described in Supporting Information: Figures S2 and S3. PCATA measurement was performed by two independent radiologists who were unaware of the patient's clinical data.

### 2.6 Ablation protocol

Non‐vitamin K antagonist oral anticoagulants (NOACs) or warfarin with a target international normalized ratio between 2.0 and 3.0 were administered in all patients. Transoesophageal echocardiography was performed to rule out thrombus before ablation.

The RFCA procedure has been described before.^13^, ^14^ Briefly, under the guidance of the CARTO system (Johnson & Johnson Medical; Biosense Webster, Inc.), circumferential pulmonary vein isolation (CPVI) was performed in AF patients. Additional linear ablation (tricuspid isthmus linear, mitral valve isthmus linear, LA roof linear) was performed in persistent AF patients. Isolation of superior vena cava (SVC) was performed if induced tachycardia from SVC or the potential of SVC was active. If AF could not be terminated after ablation, sinus rhythm was restored by cardioversion. At the end of the ablation, CPVI and bidirectional block of the lines were verified.

### 2.7 Outcomes and follow‐up

The primary outcome was the recurrence of AF after ablation during 1 year follow‐up. The definition of AF recurrence was any atrial tachyarrhythmia lasting for more than 30 seconds in electrocardiogram (ECG) or 24‐hour Holter monitoring after the 3‐month blanking period. All patients were prescribed antiarrhythmic drugs (AADs) for 3 months after ablation to prevent early recurrence. The use of AADs was subsequently determined by the physicians and patients. Patients were scheduled for follow‐up at 3‐month intervals within the first year after ablation in the outpatient setting. Each follow‐up visit included 12‐lead ECG, 24‐hour Holter monitoring, and clinical assessment. Patients who had any symptoms related to AF were required to complete an additional outpatient visit.

### 2.8 Statistical analysis

Categorical variables are presented as number (percentage) or median (Q1, Q3 quartiles) and compared between two groups by the *χ* ^2^ test. The values of the continuous variables were described as the mean ± standard deviation, and compared among groups using Student's *t*‐test and Mann−Whitney *U* test, depending on whether data were normally distributed. The relation between RCA‐PCATA and LAD‐PCATA was investigated using Pearson's correlation. Univariable and multivariable Cox proportional hazards analysis were constructed to investigate risk factors for AF recurrence. The variables with *p* < .01 in the univariable analysis and traditional risk factors for AF recurrence were used in the multivariate Cox regression analysis model, which adjusting risk factors including heart failure, prior stroke, body mass index (BMI), type of AF, duration of AF (>2 years), LA volume, and EAT volume. Receiver operating characteristic (ROC) curve was performed to determine the cut‐off value of RCA‐PCATA. The cut‐off value (−77.45 HU) was used to make the RCA‐PCATA as a categorical value. Survival analysis was visualized by Kaplan−Meier curves and significance tests between groups were conducted by the log‐rank test. Furthermore, we used restricted cubic splines (RCS) with four knots at the 5th, 25th, 75th, and 95th centiles to model the relationship between RCA‐PCATA levels and recurrence of AF, with the cut‐off value of RCA‐PCATA serving as the reference.

The performance of the prediction models for AF recurrence were compared used to determine the incremental discriminatory and reclassification performance of clinical characteristics for identify predictors of AF recurrence. The parameters included in the clinical model 1 (traditional risk model) were heart failure, prior stroke, BMI, type of AF, duration of AF (>2 years), LA volume, and EAT volume. The parameters included in the model 2 were a combination of clinical model 1 and the RCA‐PCATA as a continuous variable. Clinical model 3 was the combination of clinical model 1 and the RCA‐PCATA as a categorical variable. To further evaluate the discriminatory ability of the models, The discriminatory abilities of clinical models 2 and 3 were assessed by the reclassification performance of each model using the area under curve (AUC), relative integrated discrimination improvement (IDI), and the net reclassification improvement (NRI) values. Moreover, a subset of 50 patients were randomly selected to assess the intra‐ and interobserver reliability for the measurement of PCATA, and intra‐class correlation coefficient (ICC) was calculated for evaluation of intra‐ and interobserver agreement. Statistical analysis was performed using R language version 4.0.3 (R Foundation for Statistical Computing). A two‐sided *p*‐value of <.05 was considered statistically significant.

## 3 RESULTS

### 3.1 Patient characteristics

Five hunderd and twenty‐four patients who underwent successful RFCA for AF and pre‐ablation CCTA were screened for eligibility. Two hundred and ninety‐nine patients were included in the final analysis (Supporting Information: Figure S4). The median time from the CCTA to ablation was 3 (2−4) days.

Overall, 34.1% patients experienced recurrence of AF during 1 year follow‐up. The prevalence of recurrence was 40.9% in patients with persistent AF and 29.1% in patients with paroxysmal AF, respectively. There were not significantly different on the scan voltage 100 kV on CCTA, high‐sensitivity C‐reactive protein (hs‐CRP), linear ablation, SVC ablation, and BMI between the patients with and without recurrence. Compared to patients without AF recurrence, patients with recurrence were more likely to have heart failure, persistent AF, duration of AF >2 years. The patients with recurrence also had larger LA volume, EAT volume, RCA‐PCATA, and LAD‐PCATA. However, there was not significantly different on the value of LCX‐PCATA between two groups, as shown in Table 1.

**Table 1:** Baseline demographics and clinical characteristics.

Variables	All (*n* = 299)	Non‐recurrence (*n* = 197)	Recurrence (*n* = 102)	*p* Value
Clinical characteristics				
Age (years)	60.5 ± 11.0	60.2 ± 10.8	61.0 ± 11.3	.555
Female gender	123 (41.1%)	80 (33.5%)	43 (35.0%)	.797
BMI (kg/m)^2^	25.5 ± 3.4	25.3 ± 3.3	25.9 ± 3.6	.157
Current smoking	62 (20.7%)	38 (19.3%)	24 (23.5%)	.391
Current drinking	55 (18.4%)	36 (18.3%)	19 (18.6%)	.940
Hypertension	164 (54.8%)	104 (52.8%)	60 (58.8%)	.320
Diabetes mellitus	82 (27.4%)	55 (27.9%)	27 (26.5%)	.790
Dyslipidemia	113 (37.8%)	70 (35.5%)	43 (42.2%)	.263
Heart failure	45 (15.1%)	22 (11.2%)	23 (22.5%)	.009
Prior stroke/TIA	55 (18.4%)	31 (15.7%)	24 (23.5%)	.099
Duration of AF (>2 years)	146 (48.8%)	83 (42.1%)	63 (61.8%)	.001
Persistent AF	127 (42.5%)	75 (38.1%)	52 (51.0%)	.032
Medication				
ACEI/ARB	79 (26.4%)	47 (23.9%)	32 (40.5%)	.162
Pre‐ablation AADs	180 (60.2%)	113 (57.4%)	67 (65.7%)	.163
Statins	98 (32.8%)	60 (30.5%)	38 (37.3%)	.235
Laboratory test				
WBC (mmol/L)	6.5 ± 1.9	6.5 ± 1.8	6.6 ± 2.1	.621
PLT (μmol/L)	198.0 ± 55.5	199.0 ± 52.5	196.0 ± 61.1	.654
UA (mmol/L)	312.8 ± 89.5	313.2 ± 84.3	311.9 ± 99.2	.903
Cr (μmol/L)	75.2 ± 18.5	74.5 ± 17.1	76.8 ± 27.1	.311
hs‐CRP (mg/L)	1.4 (0.7−2.4)	1.3 (0.7−2.2)	1.4 (0.8−2.8)	.276
HbA1c (%)	6.1 ± 0.9	6.2 ± 0.9	6.1 ± 0.8	.552
Echocardiographic variables				
LVEF (%)	60.4 ± 6.6	60.6 ± 6.6	60.1 ± 6.5	.526
LA diameter	39.2 ± 6.1	38.3 ± 6.0	41.0 ± 6.0	<.001
LVEDD	47.1 ± 4.8	46.9 ± 4.6	47.5 ± 5.1	.346
CT variables				
Tube voltage of CT acquisition (kVp)				.654
100	218 (72.9%)	142 (72.1%)	76 (74.5%)	
120	81 (27.1%)	55 (27.9%)	26 (25.5%)	
LA volume	141.6 ± 47.0	135.3 ± 43.0	153.8 ± 52.4	.001
EAT volume (mL)	179.6 ± 67.0	173.6 ± 60.8	191.1 ± 76.8	.032
PCATA (HU)				
RCA	−76.9 ± 9.1	−78.4 ± 8.8	−74.0 ± 9.0	<.001
LAD	−76.2 ± 8.6	−77.0 ± 8.7	−74.8 ± 8.2	.035
LCX	−73.1 ± 7.7	−73.6 ± 7.9	−72.1 ± 7.1	.116
Linear ablation	171 (57.2%)	109 (55.3%)	62 (60.8%)	.366
SVC isolation	35 (11.7%)	22 (11.2%)	13 (12.7%)	.687

Abbreviations: AADs, antiarrhythmic drugs; ACEI, angiotensin‐converting enzyme inhibitor; AF, atrial fibrillation; ARB, angiotensin receptor blocker; BMI, body mass index; CI, confidence interval; Cr, creatinine; CT, computed tomography; EAT, epicardial adipose tissue; HbA1c, glycosylated hemoglobin; HR, hazard ratio; hs‐CRP, high‐sensitivity C‐reactive protein; HU, Hounsfield units; LA, left atrial; LAD, left anterior descending; LCX, left circumflex; LVEDD, left ventricular end‐diastolic diameter; LVEF, left ventricular ejection fraction; PCATA, pericoronary adipose tissue attenuation; PLT, platelets; RCA, right coronary artery; SVC, superior vena cava; TIA, transient ischemic attack; WBC, white blood cell.

### 3.2 PCATA characteristics

The distribution of PCATA is shown in Supporting Information: Figure S5. The mean values of LAD‐PCATA, LCX‐PCATA, and RCA‐PCATA were −76.21 HU (SD 8.59), −73.09 HU (SD 7.66), and −76.88 HU (SD 9.12), respectively. The value of RCA‐PCATA was significantly correlated with LAD‐PCATA (*r* = .687, *p* < .001), as described in Supporting Information: Figure S6. However, there were no significant association between hs‐CRP and value of RCA‐PCATA (*r* = .055, *p* = .435) and LAD‐PCATA (*r* = .160, *p* = .816). Furthermore, RCA‐PCATA (−76.64 ± 9.52 vs. −77.47 ± 8.07 HU, *p* = .475) and LAD‐PCATA (−76.38 ± 8.94 vs. −75.79 ± 7.74 HU, *p* = .588) were not significantly different between the patients with RCA dominance (*n* = 212) and with left coronary artery dominance (*n* = 87).

### 3.3 Intra‐ and interobserver reliability

Our analysis showed that the good intra‐ and inter‐reader reliability of PCATA measurements were performed in 50 randomized AF patients. The ICC of the intra‐reader reliability for LAD‐PCATA, LCX‐PCATA, and RCA‐PCATA were 0.987, 0.965, 0.984, respectively (all *p* < .001). The ICC of inter‐reader reliability for LAD‐PCATA, LCX‐PCATA, and RCA‐PCATA were 0.991, 0.977, and 0.983, respectively (all *p* < .001), as depicted in Supporting Information: Table S1.

### 3.4 The association between PCATA and recurrence of AF

**Figure 2:**
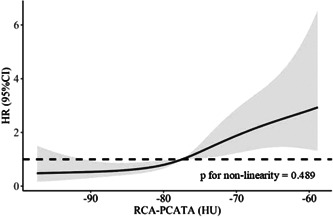
Restricted cubic spline analysis of AF recurrence risk as a function of RCA‐PCATA. The multivariable Cox analysis was adjusted for heart failure, prior stroke/TIA, BMI, type of AF, duration of AF (>2 years), LA volume, and EAT volume. AF, atrial fibrillation; BMI, body mass index; EAT, epicardial adipose tissue; LA, left atrial; PCATA, pericoronary adipose tissue attenuation; RCA, right coronary artery; TIA, transient ischemic attack.

Multivariate Cox regression analysis revealed that the value of RCA‐PCATA (HR: 1.04, 95% CI: 1.02−1.06, *p* = .001), LA volume (HR: 1.01, 95% CI: 1.00−1.01, *p* = .008), and duration of AF >2 years (HR: 1.70, 95% CI: 1.13−2.56, *p* = .011) were independently associated with AF recurrence. However, neither the value of LAD‐PCATA nor EAT volume were not independently associated with AF recurrence (Table 2). The ROC analysis revealed that the cut‐off value of RCA‐PCATA to distinguish AF recurrence was −77.45 HU. The comparison of baseline characteristics between patients with high and low value of RCA‐PCATA was described in Supporting Information: Table S2. RCA‐PCATA remained to be independently associated with AF recurrence as a categorical variable, as described in Supporting Information: Table S3. The RCS revealed that the risk of AF recurrence increased with the elevated value of RCA‐PCATA (*p* for nonlinearity = .489), as depicted in Figure 2.

**Table 2:** Risk factors for recurrence of AF by multivariable Cox regression analysis model.

Variables	Model A		Model B	
	HR (95% CI)	*p* Valu*e*	HR (95% CI)	*p* Value
Heart failure	1.50 (0.93−2.41)	.097	1.57 (0.97−2.53)	.065
Prior stroke/TIA	1.31 (0.82−2.10)	.261	1.40 (0.87−2.23)	.162
Persistent AF	1.12 (0.73−1.71)	.602	1.16 (0.76−1.77)	.492
BMI	1.04 (0.98−1.11)	.194	1.04 (0.98−1.11)	.229
Duration of AF (>2 years)	1.70 (1.13−2.56)	.011	1.75 (1.17−2.64)	.007
LA volume	1.01 (1.00−1.01)	.008	1.01 (1.00−1.01)	.007
EAT volume	1.00 (1.00−1.01)	.138	1.00 (1.00−1.01)	.163
PCATA				
RCA	1.04 (1.02−1.06)	.001		
LAD			1.02 (0.99−1.04)	.143

*Note*: Model A included heart failure, prior stroke/TIA, BMI, type of AF, duration of AF (>2 years), EAT volume, LA volume, and RCA‐PCATA. Model B included heart failure, prior stroke/TIA, BMI, type of AF, duration of AF (>2 years), EAT volume, LA volume, and LAD‐PCATA.

Abbreviations: AF, atrial fibrillation; BMI, body mass index; CI, confidence interval; EAT, epicardial adipose tissue; HR, hazard ratio; LA, left atrial; LAD, left anterior descending; PCATA, pericoronary adipose tissue attenuation; RCA, right coronary artery; TIA, transient ischemic attack.

**Figure 3:**
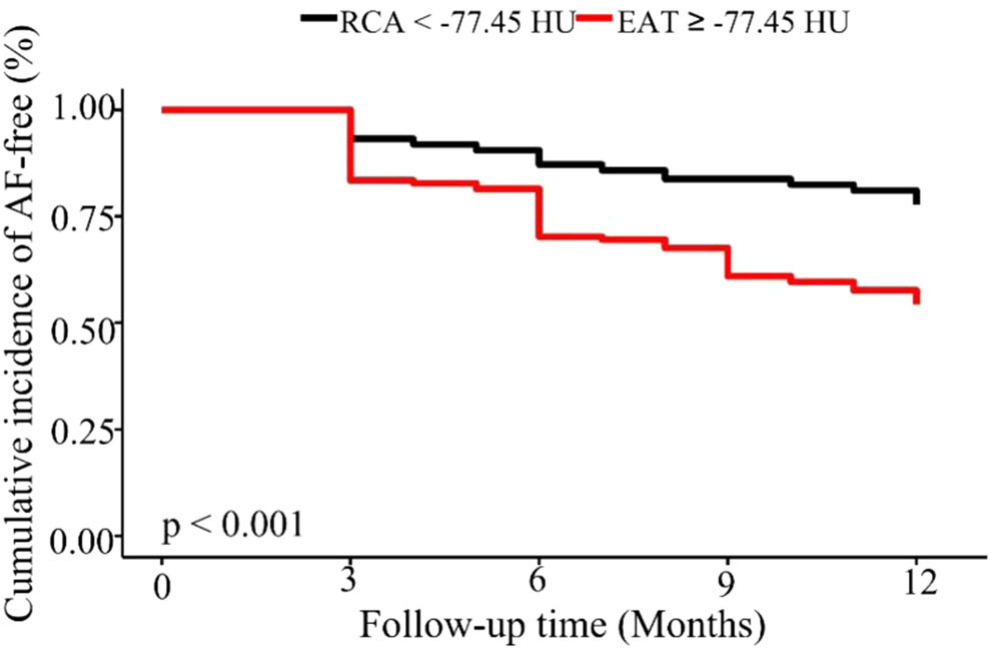
Kaplan−Meier curve for AF recurrence following radiofrequency catheter ablation. RCA‐PCATA as a categorical value is used to predict AF recurrence according to the cut‐off value (−77.45 HU). AF, atrial fibrillation; HU, Hounsfield units; PCATA, pericoronary adipose tissue attenuation; RCA, right coronary artery.

Kaplan−Meier curve analysis found that patients with high value of RCA‐PCATA had significantly increase risk of AF recurrence compared with those with low RCA‐PCATA, as described in Figure 3. Furthermore, compared to patients with the first quintile of RCA‐PCATA, the risk of AF recurrence significantly increased in patients with RCA‐PCATA within the 3th quintile, and the 4th quintile, as shown in Supporting Information: Figure S7.

### 3.5 Comparison of the predicting models for AF recurrence

The addition of RCA‐PCATA as a continuous variable significantly improved the predictive ability for AF recurrence compared with the clinical model 1 (AUC: 0.724 vs. 0.686, *p* = .024). The incremental reclassification efficacy for predicting AF recurrence was significantly improved by adding RCA‐PCATA (relative IDI: 0.043, *p* = .006; continuous NRI: 0.521, *p* < .001). Moreover, the efficacy for predicting AF recurrence was also significantly improved by adding RCA‐PCATA as a categorical variable, as described in Supporting Information: Table S4.

## 4 DISCUSSION

This is a pilot study to investigate the association between PCATA and AF recurrence after RFCA. Our main findings revealed that RCA‐PCATA was an independent predictor for AF recurrence. Our study may help physicians identify high risk of AF recurrence patients following catheter ablation.

The high level of inflammation could regulate calcium homeostasis and connexins of cardiac myocyte, all of which could promote AF development.^6^ Adipose tissue attenuation may reflect the change of inflammatory levels.^15^ The association between PCATA and vascular inflammation is confirmed by the biopsy tissue study in patients with cardiac surgery.^16^ Normal PCAT exerts an anti‐inflammatory function, which is shown by the aggregation of T‐lymphocytes and the secretion of cytokines. The high‐level attenuation of PCAT indicates the change in intracellular lipid accumulation, which caused by chronic and early inflammation.^17^ PCAT dysfunction is related to inflammation and oxidative stress by the infiltration of immune cell. The proinflammatory environment also enhances the infiltration of additional adipose tissue immune cells, forming a vicious cycle.^18^ Therefore, a routine, noninvasive method to quantify the degree of pericoronary inflammation is highly credible.

Pericoronary adipose tissue could secrete bioactive substances and plays an important role in vasoconstriction and dilatation, and smooth muscle cell proliferation.^19^ PCATA has been shown to be an independent predictor of prognosis in patients with coronary artery disease.^10^, ^20^ PCATA can be used to monitor the inflammation change and track response to anti‐inflammatory therapy.^21^ One study found that PCATA, which represents pericoronary inflammation, is an independent predictor for AF recurrence after ablation with second‐generation cryoballoon.^22^ Our study was performed in patients with radiofrequency ablation, rather cryoablation. Our study also investigated the effect of PCATA in three different coronary vessels on recurrence after AF ablation, rather than the average PCATA in three vessels. Another study revealed that LCX‐PCATA is an independent predictor for AF recurrence following ablation in 189 AF patients. However, there were 107 patients with coronary heart disease enrolled in this study. It is well known that coronary heart disease has a significant impact on PCATA, which may lead to a bias in the study results. Our study included 299 patients with AF ablation and excluded patients with coronary artery disease according to CCTA or coronary angiography. We found that RCA‐PCATA is an independent risk factor for recurrence of AF following radiofrequency ablation. We consider that the high level of CT attenuation may reflect adverse outcomes due to tissue activity and inflammation elevated, which prevents the formation of the ideal ablation range by altering the intrinsic characteristics of the tissue, including impedance and conductivity.^23^ Our study suggested that PCATA surrounding the proximal RCA may be identify recurrence of high risk in AF ablation patients.

The previous study revealed that RCA‐PCATA is independently associated with adverse cardiac events rather than LAD‐PCATA in diabetes patients.^24^ Another study extended these findings, found that high PCATA in the proximal RCA is associated with increased cardiac mortality.^10^ In a cross‐sectional study about different coronary artery disease stages, RCA‐PCATA was unaffected by the distribution of culprit lesions, suggesting that the proximal RCA has been used as a biomarker of global pericoronary inflammation.^25^ Our study revealed that RCA‐PCATA and LAD‐PCATA were not influenced by whether RCA dominance. These vascular based variations in PCATA may be attributed to differences in anatomy and surrounding tissues. PCATA is more prevalent around RCA than in the left coronary artery. The proximal RCA is surrounded by the largest volume of adipose tissue and is not interfering, such as side branches, coronary veins, or myocardium.^25^ The pericoronary fat attenuation signal around the left coronary artery close to the lung may be affected by the lung.^26^ RCA‐PCATA may explain the better prognostic value for clinical outcomes, compared with the left coronary artery. Our study also suggested that the RCA‐PCATA with conventional clinical factors improved the incremental efficacy of predicting the incidence of AF recurrence. RCA‐PCATA may be a more reliable measure on CCTA about the global state inflammation of the coronary vasculature. The PCATA index in CCTA reporting might be more meaningful for assessing patients' risk‐stratification.

EAT is a unique type of visceral adipose tissue located between the myocardium and visceral pericardium, without an intervening fascial plane.^27^ Previous studies revealed that EAT volume is a valuable predictor for the new onset of AF and recurrence of AF following ablation.^28^ However, our study revealed that the total EAT volume was not an independent factor for the recurrence of AF. Research suggested that the quality of LA adipose tissue, but not total EAT volume is an independent predictor of AF recurrence after ablation.^29^, ^30^ The high level attenuation of adipose tissue in the posterior of LA may indicate the high‐level local inflammation and is independently associated with AF recurrence.^8^ Adipose tissue attenuation is more reliably than fat volume because it does not depend on any anatomical markers established manually and subjectively by the researcher performing the measurement.^9^ Unfortunately, we did not measure the attenuation of EAT surrounding the LA. PCAT is considered to be part of EAT, which contains anti‐inflammatory and antioxidant substances as well‐as inflammatory components and therefore plays a key role in both cardiovascular homeostasis and cardiovascular disease.^31^ We postulated that PCATA is a surrogate. PCATA measurement is simple and convenient. PCATA can be measured directly on CCTA, rather than the need for other complex radiomic features and complex modeling software.^32^

Some limitations of the study need to be acknowledged. First, this was a retrospective, single‐center study with a limited sample size, and selection bias may exist. Although the follow‐up was prospective and detailed, and improving the quality of data. Second, some patients with AF recurrence might have been missed for asymptomatic arrhythmia, although we encouraged patients to contact the physician in the case of suspected recurrence symptoms. Third, we did not have the local or systemic total markers of inflammation data to support the association with PCATA, as hs‐CRP was only available in 69.2% AF patients. Finally, PCATA data measurement through semiautomated software may still be biased, although we use well‐established software to measure PCATA and the reliability of the measured data has been confirmed in several previous studies.^24^, ^33^

## 5 CONCLUSIONS

The high level of RCA‐PCATA was an independent predictor for AF recurrence following RFCA. The PCATA may help to recognize high recurrence risk following ablation in AF patients. Further larger cohort studies to validate these findings are certainly needed to provide us with more consistent data.

## AUTHOR CONTRIBUTIONS

Zhe Wang, Ying‐Wei Chen, and Yi‐Hong Sun conceived and designed the study. Zhe Wang, Jia‐Wei Chen, Li‐Chen Ren, and He‐He Guo participated in the acquisition of data. Zhe Wang, Jia‐Wei Chen, and He‐He Guo analyzed the data. He‐He Guo and Xiao‐Jie Chen gave advice on methodology. Zhe Wang and Yi‐Jia Wang drafted the manuscript. Yi‐Hong Sun revised the manuscript. All authors read and approved the final manuscript.

## CONFLICT OF INTEREST STATEMENT

The authors declare no conflict of interest.

## Supporting information

Supporting information.

## Data Availability

The data supporting the findings of this study are available on request.
