# Supplementary material for: Impact of pericoronary adipose tissue attenuation on recurrence after radiofrequency catheter ablation for atrial fibrillation
Source: Clin Cardiol. 2023 Jul 12;46(10):1244–52. doi: 10.1002/clc.24081 (PMC10577529; doi:10.1002/clc.24081)
Supplement: Supplementary file 1 — Supporting information. [file CLC-46-1244-s001.docx]

**FIGURE S1.** Example of coronary computed tomography angiography measurement of epicardial adipose tissue volume (red arrow) in AF patients. AF, atrial fibrillation.

**
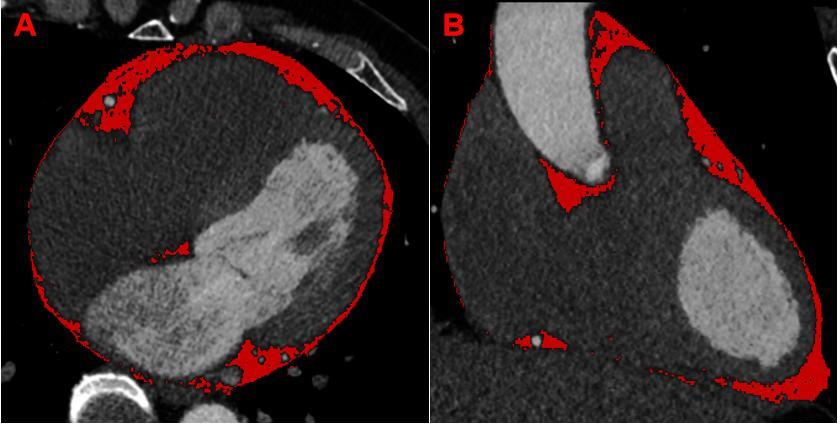
**

**FIGURE S2.** Semi-automated PCATA of LAD analysis on coronary computed tomography angiography. (A) Three-dimensional reconstruction of all three major epicardial coronary vessels; (B) The PCAT phenotyping around the proximal LAD 40 mm segment was traced for the vessel; (C) The color map of coronary computed tomography for PCATA in the cross-sectional view. PCAT is defined as fat within a radial distance equal to the diameter of the vessel. AF, atrial fibrillation; HU, Hounsfield unit; LAD, left anterior descending; LM, left main; PCAT, pericoronary adipose tissue; PCATA, pericoronary adipose tissue attenuation.


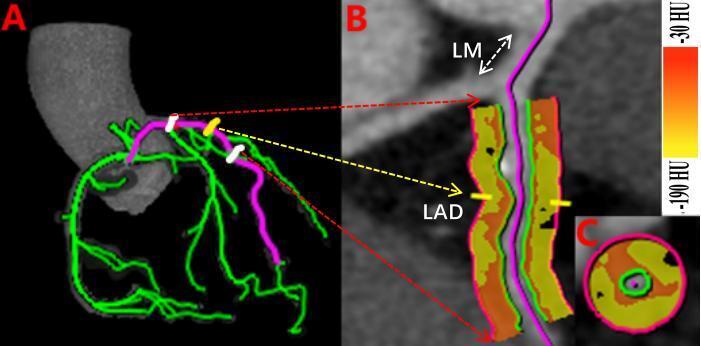


**FIGURE S3.** Semi-automated PCATA of LCX analysis on coronary computed tomography angiography. **(**A) Three-dimensional reconstruction of all three major epicardial coronary vessels; (B) An example of PCAT phenotyping around the proximal LCX 40 mm segment was traced for the vessel; (C) The color map of coronary computed tomography for PCATA in the cross-sectional view. PCAT was defined as fat within a radial distance equal to the diameter of the vessel. AF, atrial fibrillation; HU, Hounsfield unit; LCX, left circumflex; LM, left main; PCAT, pericoronary adipose tissue; PCATA, per coronary adipose tissue attenuation.


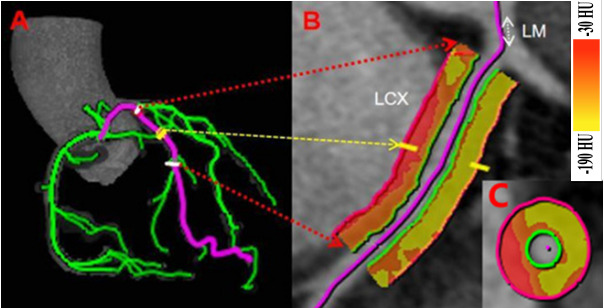


**FIGURE S4.** The flowchart of the study cohort. AF, atrial fibrillation; CCTA, coronary computed tomography angiography; RFCA, radiofrequency catheter ablation.

**
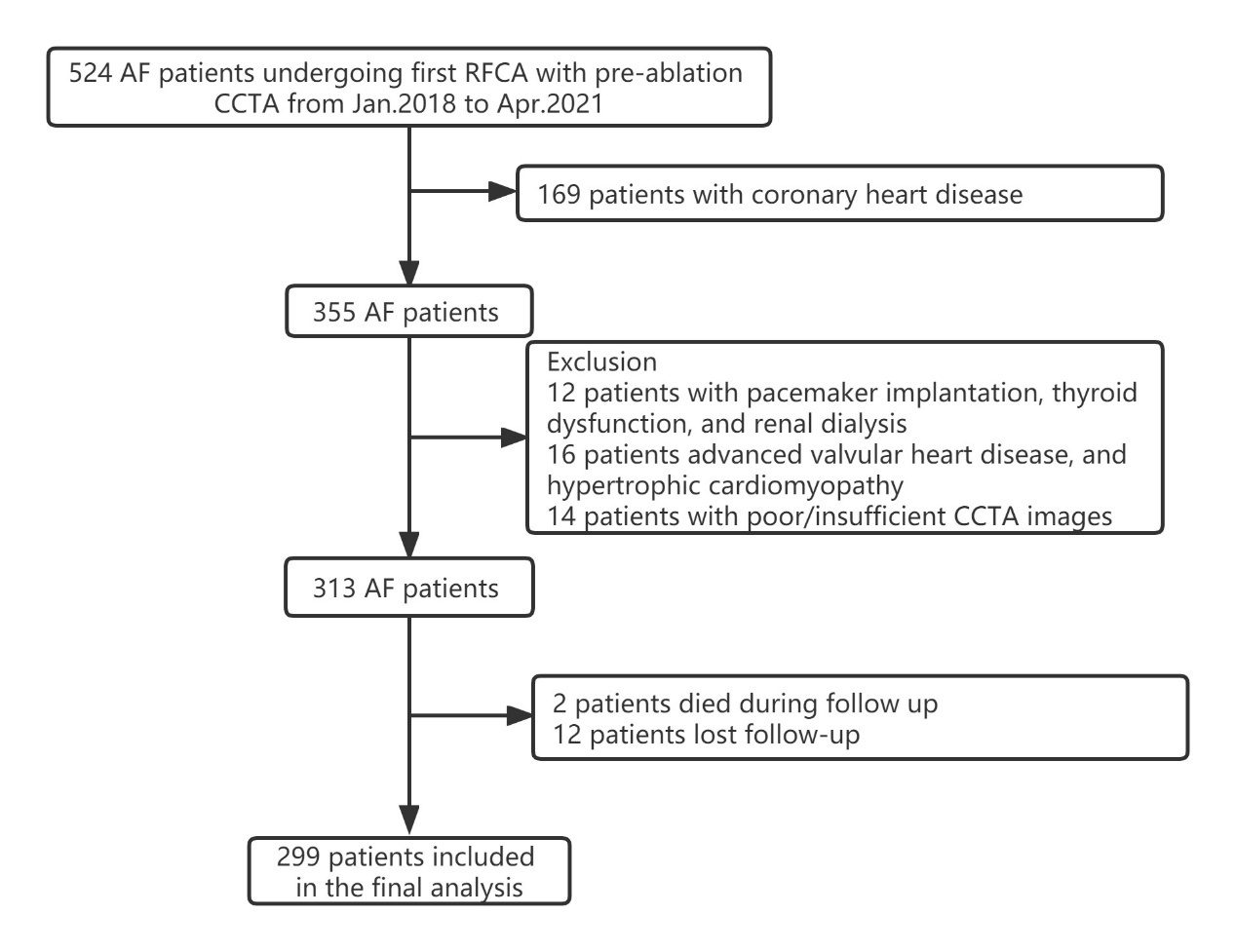
**

**FIGURE S5.** Distribution of PCATA in AF patients according to three main coronary arteries. Different color dots represent measured PACTA; Top of box, 75th percentile; horizontal line, 50th percentile (median); bottom of the box, 25th percentile; whiskers, maximum and minimum PCATA except for outliers, respectively. AF, atrial fibrillation; HU, Hounsfield units; LAD, left anterior descending; LCX, left circumflex; PCATA, pericoronary adipose tissue attenuation; RCA, right coronary artery.

**
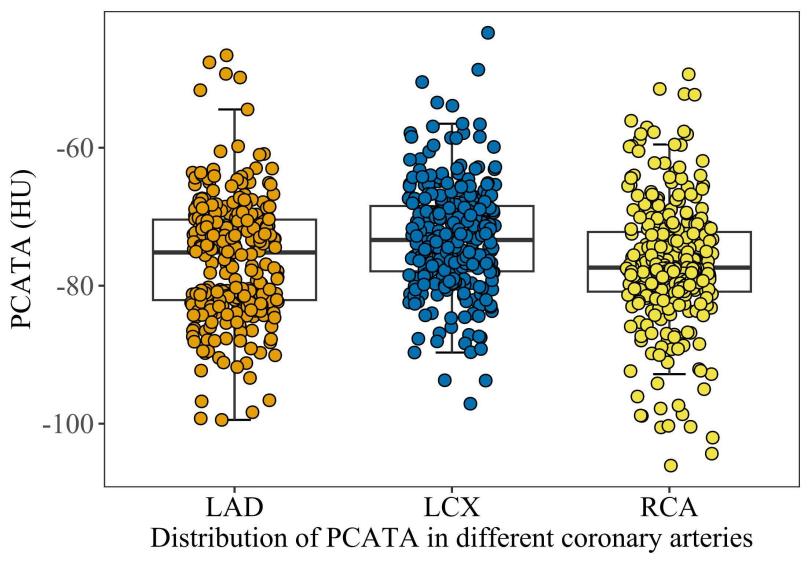
**

**FIGURE S6.** The correlations between RCA-PCATA and LAD-PCATA. LAD, left anterior descending; HU, Hounsfield units; PCATA, pericoronary adipose tissue attenuation; RCA, right coronary artery.


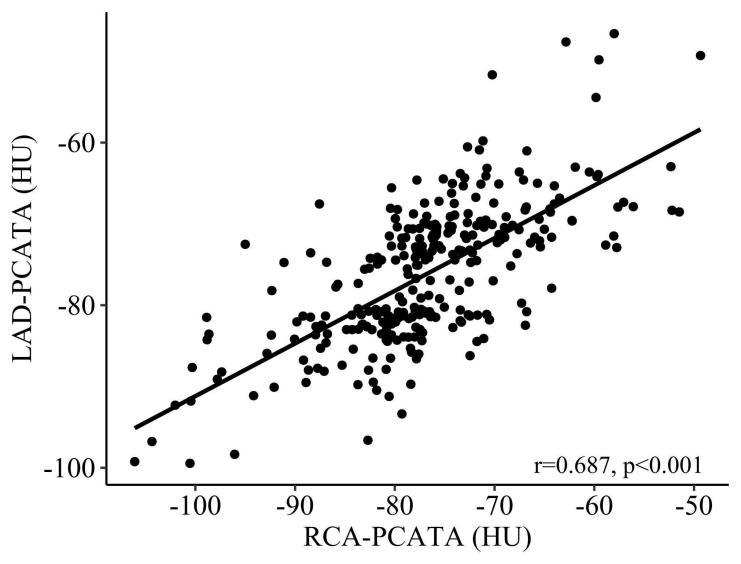


**FIGURE S7.** The hazard ratio for AF recurrence by interquartile of RCA-PCATA. Filled circles and vertical lines indicate the HR and 95% CI for interquartile 2 to 4 of RCA-PCATA; relative to IQR 1. IQR 1 (RCA-PCATA ≤ -80.87 HU); IQR 2 (-80.87 HU < RCA-PCATA ≤ -77.38 HU); IQR 3 (-77.38 HU < RCA-PCATA ≤ -72.20 HU); IQR 4 (RCA-PCATA > -72.20 HU). AF, atrial fibrillation; CI, confidence interval; HR hazard ratio; HU, Hounsfield units; IQR, interquartile; PCATA, pericoronary adipose tissue attenuation; RCA, right coronary artery.


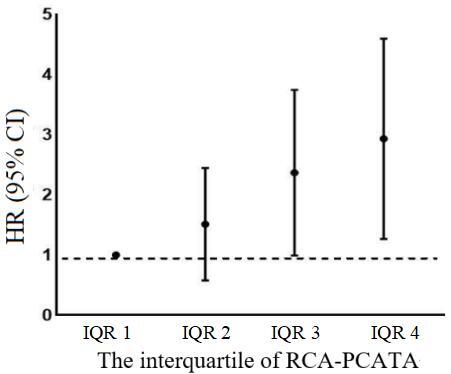


**TABLE S1.** Intra- and interobserver reliability in PCATA measurement.

|  | ICC (95% CI) | *p*-value |
| --- | --- | --- |
| Intra-observer reliability in PCATA measurement | | |
| RCA-PCATA | 0.984 (0.843-0.995) | <0.001 |
| LAD-PCATA | 0.987 (0.892-0.996) | <0.001 |
| LCX-PCATA | 0.965 (0.898-0.984) | <0.001 |
| Inter-observer reliability in PCATA measurement | | |
| RCA-PCATA | 0.983 (0.967-0.991) | <0.001 |
| LAD-PCATA | 0.991 (0.984-0.995) | <0.001 |
| LCX-PCATA | 0.977 (0.941-0.989) | <0.001 |

Abbreviations: CI, confidence interval; ICC, intraclass correlation coefficient; LAD, left anterior descending; LCX, left circumflex; PCATA, pericoronary adipose tissue attenuation; RCA, right coronary artery.

**TABLE S2.** Comparison of baseline characteristics between AF patients with high and low PCATA of RCA.

| Variable | High RCA-PCATA | Low RCA-PCATA | *p*-value |
| --- | --- | --- | --- |
| Patients | 151 | 148 |  |
| Clinical characteristics |  |  |  |
| Age, years | 59.9 ± 10.8 | 61.0 ± 11.2 | 0.377 |
| Female gender | 61 (40.4%) | 62 (41.9%) | 0.793 |
| BMI, kg/m^2^ | 25.0 ± 3.3 | 26.0 ± 3.4 | 0.015 |
| Current smoking | 34 (22.5%) | 28 (18.9%) | 0.443 |
| Current drinking | 31 (20.5%) | 24 (16.2%) | 0.336 |
| Hypertension | 84 (55.6%) | 80 (54.1%) | 0.784 |
| Diabetes mellitus | 45 (29.8%) | 37 (25.0%) | 0.352 |
| Dyslipidemia | 52 (34.4%) | 61 (41.2%) | 0.227 |
| Heart failure | 26 (17.2%) | 19 (12.8%) | 0.290 |
| Prior stroke/TIA | 29 (19.2%) | 26 (17.6%) | 0.715 |
| Duration of AF (>2 years) | 84 (55.6%) | 62 (41.9%) | 0.018 |
| Persistent AF | 74 (49.0%) | 53 (35.8%) | 0.021 |
| Medication |  |  |  |
| ACEI/ARB | 43 (28.5%) | 36 (24.3%) | 0.416 |
| Pre-ablation AADs | 92 (60.9%) | 88 (59.5%) | 0.795 |
| Statins | 41 (27.2%) | 57 (38.5%) | 0.036 |
| Laboratory test |  |  |  |
| hs-CRP, mg/l | 1.4 (0.8, 2.7) | 1.2 (0.7, 2.2) | 0.115 |
| HbA1c, % | 6.1 ± 0.8 | 6.1 ± 0.9 | 0.845 |
| LVEF, % | 60.4 ± 7.1 | 60.5 ± 6.0 | 0.998 |
| LA diameter, mmm | 38.7 ± 6.2 | 39.8 ± 6.0 | 0.121 |
| LVEDD, mmm | 47.5 ± 5.2 | 46.8 ±4.3 | 0.201 |
| CT variables |  |  | 0.586 |
| 100Kvp | 108 (71.5%) | 110 (74.3%) |  |
| 120Kvp | 43 (28.5%) | 38 (25.7%) |  |
| LA volume, ml | 144.7 ± 43.8 | 138.5 ± 49.9 | 0.249 |
| EAT volume, ml | 176.4 ± 65.6 | 182.9 ± 68.5 | 0.406 |

Note: Continuous data are presented as means ± standard deviation (SD) or median (inter-quartile range), and categorical data were shown as n (%). The high and low RCA-PCATA groups were divided according to the best cut-off values of RCA-PCATA (−77.45 HU).

Abbreviations: AADs, anti-arrhythmic drugs; AF, atrial fibrillation; ACEI, angiotensin-converting enzyme inhibitor; ARB, angiotensin receptor blocker; BMI, body mass index; Cr, creatinine; EAT, epicardial adipose tissue; HbA1c, glycosylated hemoglobin; hs-CRP, high-sensitivity C-reactive protein; LA, left atrial; LVEDD, left ventricular end-diastolic diameter; LVEF, left ventricular ejection fraction; PCATA, pericoronary adipose tissue attenuation; RCA, right coronary artery; TIA, transient ischemic attack.

**TABLE S3.** Risk factors for AF recurrence by multivariable Cox regression analysis.

| Variable | HR (95%CI) | | *p*-value |
| --- | --- | --- | --- |
| Heart failure | | 1.59 (0.98~2.56) | 0.059 |
| Prior stroke/TIA | | 1.36 (0.85~2.17) | 0.196 |
| Persistent AF | | 1.11 (0.73~1.71) | 0.619 |
| BMI | | 1.05 (0.98~1.12) | 0.150 |
| Duration of AF (>2 years) | | 1.72 (1.14~2.58) | 0.009 |
| LA volume | | 1.01 (1.00~1.01) | 0.009 |
| EAT volume | | 1.00 (1.00~1.01) | 0.185 |
| RCA-PCATA as a categorical variable | | 2.22 (1.45~3.39) | <0.001 |

Note: The model included heart failure, prior stroke/TIA, BMI, type of AF, duration of AF (>2 years), LA volume, EAT volume, and RCA-PCATA as a categorical variable; Patients with AF are divided into two groups according to the best cut-off attenuation value of RCA-PCATA (−77.45 HU).

Abbreviations: AF, atrial fibrillation; BMI, body mass index; CI, confidence interval; EAT, epicardial adipose tissue; HR, hazard ratio; LA, left atrial; PCATA, pericoronary adipose tissue attenuation; RCA, right coronary artery; TIA, transient ischemic attack.

**TABLE S4.**Comparison of efficacy of models for predicting AF recurrence.

| Models |  |  | Relative IDI | | | | Continuous NRI | | |
| --- | --- | --- | --- | --- | --- | --- | --- | --- | --- |
|  | AUC | *p*-value | IDI (95% CI) | *p*-value | | NRI (95% CI) | | *p*-value | |
| Clinical model 1(Traditional risk model) | 0.686 | Reference | Reference | |  | | Reference | |  |
| Clinical model 2  (Clinical model 1 + -RCA-PCATA as a continuous variable) | 0.724 | 0.024 | 0.043 (0.014~0.083) | | 0.006 | | 0.521 (0.293~0.748) | | <0.001 |
| Clinical model 3  (Clinical model 1 + RCA-PCATA as a categorical variable) | 0.736 | 0.020 | 0.050 (0.012~0.109) | | 0.008 | | 0.377 (0.141~0.612) | | 0.002 |

Note: Clinical model 1 included heart failure, prior stroke/TIA, BMI, type of AF, duration of AF (>2 years), EAT volume, and LA volume; Clinical model 2 included a combination of clinical model 1 and RCA-PCATA as a continuous variable; Clinical model 3 included a combination of clinical model 1 and RCA-PCATA as a categorical variable.

Abbreviations: AF, atrial fibrillation; AUC, area under curve; BMI, body mass index; CI, confidence interval; EAT, epicardial adipose tissue; HR, hazard ratio; IDI, integrated discrimination improvement; LA, left atrial; NRI, net reclassification improvement; PCATA, pericoronary adipose tissue attenuation; RCA, right coronary artery; TIA, transient ischemic attack.
